# Distributed Rate-Control and Delay-Guaranteed Scheduling in MR-MC Wireless Mesh Networks

**DOI:** 10.3390/s19225005

**Published:** 2019-11-16

**Authors:** Liang Li, Xiongwen Zhao, Suiyan Geng, Yu Zhang

**Affiliations:** School of Electrical and Electronic Engineering, North China Electrical Power University, Beijing 102206, China

**Keywords:** scheduling algorithm, multi-radio multi-channel (MR-MC), wireless mesh networks (WMNs)

## Abstract

Wireless mesh networks (WMNs) can provide flexible wireless connections in smart city, Internet of Things (IoT), and device-to-device (D2D) communications. The performance of WMNs can be greatly enhanced by adopting the multi-radio multi-channel (MR-MC) technique, which enables a node to communicate with more nodes simultaneously. However, increasing the number of data flows will result in network congestion and longer end-to-end delays. In this paper, a distributed rate-control and delay-aware (DRDA) scheduling algorithm is proposed based on a multidimensional conflict graph. To satisfy the arrival rate and delay constraints of a flow, two virtual queues are constructed. All the actual and virtual queues are stabilized by the Lyapunov drift optimization method. The scheduling policy of each flow is optimized only based on the local information. The simulation results show that our proposed algorithm can maintain the stability of all the queues and strictly satisfy the arrival rate and delay constraint of each flow in the network as well.

## 1. Introduction

By increasing the dimensions of the radio interfaces and channels of a node, the multi-radio multi-channel (MR-MC) technique can significantly improve the capacity of wireless mesh networks (WMNs) [1,2,3,4,5]. MR-MC WMNs constitute an attractive complementary or even standalone solution for vehicle-to-vehicle (V2V), device-to-device (D2D), and unmanned aerial vehicle (UAV) communications to provide access to rural areas with little wired infrastructure or to enhance connectivity in highly dense metropolitan areas [6,7,8,9,10,11].

The problem of minimizing congestion and end-to-end delay in traffic scheduling, which is of fundamental importance for achieving superior performance while maintaining network connectivity, has attracted much attention recently [12,13]. Many scheduling algorithms have been proposed for single-radio single-channel (SR-SC) networks. A max-weight-based scheduling algorithm was proposed in [14], where the stability of the queue length was transformed into a convex optimization problem with the objective being to maximize the average throughput. The epidemic theory and truthful mechanism were adopted in scheduling algorithms to satisfy the delay constraint in [15,16], respectively. A scheduling algorithm based on the physical-ratio-K (PRK) interference model was proposed in [17], where the controllers identify the interference parameter in the PRK model as a minimum-variance regulation control problem and adapt its PRK model parameters to guarantee the link reliability desired. There are some works applying the back-pressure method to schedule flows for data transmission [18,19]. Scheduling based on the back-pressure algorithm is equivalent to solving the problem of a maximum weighted independent set (MWIS), which has been proven to be NP-hard. Several suboptimal scheduling algorithms have been proposed to approximate throughput optimality with low computation complexity [20,21,22,23]. A popular algorithm is greedy maximal scheduling (also known as the longest-queue-first policy), which is based on the greedy MWIS algorithm, in [20]. The Lyapunov-based approach is a novel method for optimizing a queue scheduling algorithm [24,25,26]. The throughput and reliability of a network with delay constraints were optimized through the Lyapunov method in [27,28], respectively. In [29], a Lyapunov-based buffer management strategy was designed to allocate the buffer recourses at each queue with video streaming.

Since centralized scheduling policies are not favored in large-scale sensor networks, especially those networks with some mobile and multi-hop nodes [30], distributed scheduling policies have been studied in the open literature. The authors of [31] studied the tradeoff between centralized and distributed approaches by way of the channel state information (CSI), and the point at which distributed scheduling outperforms centralized scheduling was characterized. Distributed graph routing and autonomous scheduling (DiGS) was proposed in [32], where the devices can compute their own transmission schedules autonomously based on the graph routes. Due to the scheduling conflicts among different types of traffic being eliminated by the graph routes, DiGS can provide short end-to-end latency. In [33], a local voting scheduling algorithm was proposed in which the load was defined as the ratio of the queue length to the number of allocated slots. All the loads were semi-equalized through slot reallocation based on local information exchange. The interference in a multi-hop network is more severe than that in a regular one due to the simultaneously transmitting links in the network. There are some scheduling algorithms which exploit a physical layer model to optimize the network throughput, for example, [34] presented a class of localized scheduling algorithms with a provable throughput guarantee subject to physical interference constraints, while [35,36] optimized distributed link scheduling under the signal to interference plus noise ratio (SINR) model.

In MR-MC networks, with significantly increased network dimension, link scheduling is often coupled with radio/channel assignment. There have been only a few studies on designing and analyzing the performance of distributed scheduling policies in MR-MC networks [2]. In [37], a distributed maximal scheduling policy was applied in MR-MC networks based on link–channel pairs (LCPs), where a physical link was split into several LCPs, with each LCP maintaining a queue to be considered in the maximal scheduling. A systematic approach that transforms an MR-MC network into an equivalent virtual SR-SC network through a tuple-based multidimensional conflict graph (MDCG) was proposed in [2]. Furthermore, cross-layer framework distributed scheduling and delay-aware routing based on an MDCG in multi-hop MR-MC networks was proposed in [38]. However, the distributed scheduling algorithm in [38] only considers the rate control of each node, which neglects the optimization of the scheduling decisions for multiple flows in each node.

Different from the aforementioned algorithms, we not only incorporate novel virtual queue structures to share the burden of actual packet queue backlogs through the virtual queues in an attempt to guarantee the delay performance and finite buffer sizes, but we also construct a scheduling controller to decide which flow needs to be scheduled at this moment, and the transmission rate of this flow is also calculated in a distributed way. The Lyapunov optimization method is adopted to maximize the data rate of the source node with an end-to-end delay constraint, actual queue backlogs, interference from neighbor links, and any other network limitations. The main contributions of this paper are summarized as follows:

- A distributed rate-control and delay-guaranteed scheduling algorithm for MR-MC WMNs is proposed. The scheduling policy and scheduling rate of a link are optimized in a distributed way.
- Two virtual queues are constructed to satisfy the data rate and delay constraint, respectively. The Lyapunov drift optimization method is adopted to maintain the queue stability and maximize the data rates of all the flows given the limitations in the MR-MC WMNs.
- A weighted scheduling metric is designed which is only based on the local information. The transmission rates of the scheduled flows for each link can also be calculated in a distributed way.

The remainder of this paper is organized as follows. The system model based on an MDCG and the queue models are developed in Section 2. In Section 3, we introduce the Lyapunov drift optimization algorithm and analyze its performance. The distributed rate-control and delay-guaranteed scheduling algorithm is proposed in Section 4, and simulation results are given in Section 5. Section 6 concludes the article.

## 2. System Model

### 2.1. A Tuple-Based Link Model of MR-MC Networks

We consider an MR-MC multi-hop WMN as an undirected graph $G_{p}(N,{ℒ}_{p})$ where the sets of nodes and physical links are denoted by N and ${ℒ}_{p}$, respectively. Each node n∈N is equipped with a set of radio interfaces $r_{n}$, each of which can operate on a set of orthogonal channels e. Define tuple $p_{n}=(n,r_{n},e_{r_{n}})$ as a vertex in the multidimensional conflict graph, which indicates that the radio interface $r_{n}\in r_{n}$ of node n∈N operates on channel $e_{r_{n}}\in e$. Let the tuple-link $l=(p_{m},p_{n})=((n,r_{n},e_{l}),(m,r_{m},e_{l}))$ represent the transmission from interface $r_{n}$ of node n to interface $r_{m}$ of node m. Two radio interfaces can communicate with each other if, and only if, they work on the same channel, i.e., $e_{l}=e_{r_{n}}=e_{r_{m}}$. Assuming that T and ℒ are the sets of tuples and tuple-links, respectively, then the whole network can be mapped as a tuple-based graph G(T,ℒ). For convenience, the terms “tuple-link” and “link” are used interchangeably in this paper. Referring to the protocol interference model in [39], making the assumption that the interference range is equal to the communication range, collision occurs when two links share the same radios at the sending or receiving node or when the two links are within communication range of each other and with the same channel. For each link l∈ℒ, we define $I_{l}$ as the set of all interference links of l.

**Figure 1:**
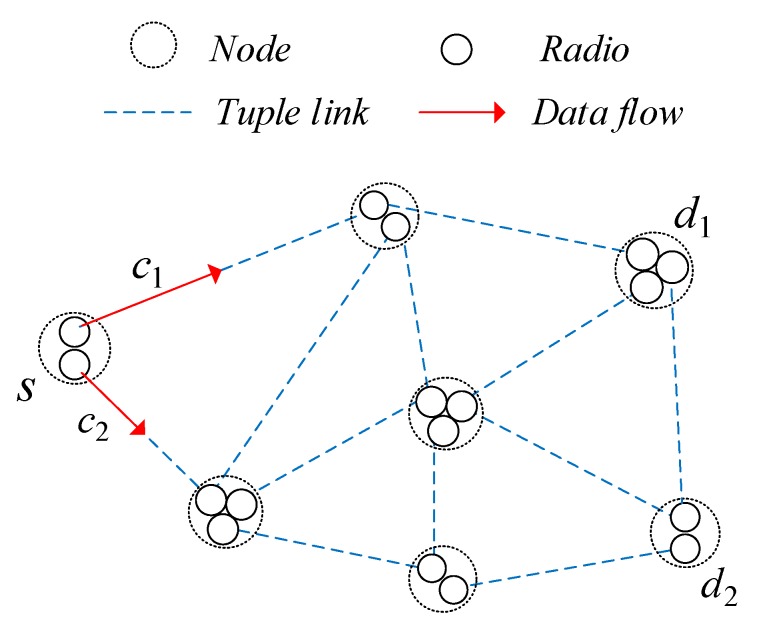
The multicommodity flow problem based on a tuple-based link model.

In this work, we study the multicommodity flow problem in which a set of flows F is injected into the network. The minimum arrival rate required for flow c∈F is denoted $a_{c}$. We denote the source node that generates flow c by s(c), s(c)∈N. Every node in the network is equipped with two or three radios, and more than three orthogonal channels are available in the network. The nodes choose channels and the next hop according to a certain channel assignment and routing algorithm, which is not the focus here and can be found in [40]. Taking the simple network shown in Figure 1 as an example, the source node s generates two commodity flows $c_{1},c_{2}\in F$, each of which has a different destination node, $d_{1}$ and $d_{2}$, respectively. The flows may go by way of multiple hops to reach the destination, so our goal is to design a proper scheduling policy for each node to deliver the packets to their corresponding destinations at the maximal throughput rate with an end-to-end delay limitation. The key notation in this paper is summarized in Table 1. E{·} and |·| are the expectation and absolute value, respectively. The operator ${\left[x\right]}^{+}$ is defined as ${\left[x\right]}^{+}=\max\{x,0\}$.

### 2.2. Queue Model

**Figure 2:**
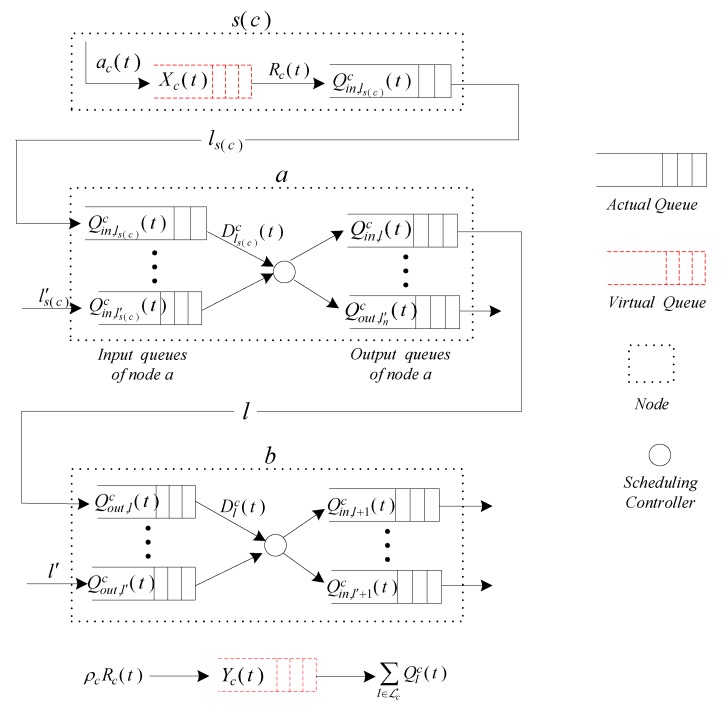
Actual and virtual queue model.

In this system, we consider the queue model which contains two types of queues, namely, actual queues and virtual queues. As shown in Figure 2, each link contains two actual queues; one is the input queue for incoming packets, and the other is the output queue for departing packets. We define $Q_{l}^{c}(t)$ as the queue backlog of link l with flow c at time *t*. $Q_{in,l}^{c}(t)$ and $Q_{out,l}^{c}(t)$ represent the backlogs of the input and output queues for link *l* with flow c at time *t*, respectively. The relationship of $Q_{l}^{c}(t)$, $Q_{in,l}^{c}(t)$, and $Q_{out,l}^{c}(t)$ can be expressed as

(1) $$Q_{l}^{c}(t)=Q_{in,l}^{c}(t)+Q_{out,l}^{c}(t),\;\forall c\in F,l\in {ℒ}_{c},$$

where $Q_{l}^{c}(t)$ can be calculated as

(2) $$Q_{l}^{c}(t+1)={\left[Q_{l}^{c}(t)-D_{l}^{c}(t)\right]}^{+}+D_{l-1}^{c}(t),\;\forall c\in F,l\in {ℒ}_{c},l\neq l_{s(c)},$$

where the terms $D_{l}^{c}(t)$ and $D_{l-1}^{c}(t)$ represent the scheduling policies of links l and (l−1) of flow c, respectively, with $l,\;(l-1)\in {ℒ}_{c}$. The scheduling policy of each link is calculated by the scheduling controller in each node, which consists of two parts. One part is to determine which flow should be scheduled, and the other is to determine the scheduling rate of the flow scheduled. The scheduling policy $D_{l}^{c}(t)$ is expressed as

(3) $$D_{l}^{c}(t)=\delta_{l}^{c}(t)\mu_{l}^{c}(t),\;\forall c\in F,l\in {ℒ}_{c}$$

where $\delta_{l}^{c}(t)$ represents the scheduling decision of link l with flow c, and $\delta_{l}^{c}(t)=1$ indicates that link l with flow c will be scheduled in the next time period, while $\delta_{l}^{c}(t)=0$ indicates that link l with flow c will not be scheduled. $\mu_{l}^{c}(t)$ denotes the scheduling rate of link l with flow c. The values of $\delta_{l}^{c}(t)$ and $\mu_{l}^{c}(t)$ are calculated by the scheduling controller in a distributed way, which will be discussed in Section 4.

The queue model of the source link is different from that of other links because its data input rate $R_{c}(t)$ is a virtual rate of flow c. Let s(c) denote the source node with flow c. $l_{s(c)}$ is the source (first) link of flow c. The queue backlog of source link $l_{s(c)}$ can be calculated by

(4) $$Q_{l_{s(c)}}^{c}(t+1)={\left[Q_{l_{s(c)}}^{c}(t)-D_{l_{s(c)}}^{c}(t)\right]}^{+}+R_{c}(t),\;\forall c\in F,l_{s(c)}\in {ℒ}_{c}$$

where $D_{l_{s(c)}}^{c}(t)$ is the scheduling policy of source link $l_{s(c)}$.

Now we construct two virtual queues, namely, the virtual service queue $X_{c}(t)$ at the source node and the virtual delay queue $Y_{c}(t)$ for all the links of flow c. To satisfy the minimum arrival data rate requirement from the transport layer, the virtual queue $X_{c}(t)$ is constructed as

(5) $$X_{c}(t+1)={\left[X_{c}(t)-R_{c}(t)\right]}^{+}+a_{c}(t),\;\forall c\in F$$

where $a_{c}(t)$ is the minimum arrival rate of flow c. To satisfy the delay requirements of flow c, the virtual queue $Y_{c}(t)$ is constructed as

(6) $$Y_{c}(t+1)={\left[Y_{c}(t)-\rho_{c}R_{c}(t)\right]}^{+}+\sum_{l\in {ℒ}_{c}}Q_{l}^{c}(t),\;\forall c\in F$$

where $\rho_{c}$ is the maximum delay constraint of flow c, $\rho_{c}R_{c}(t)$ is the virtual backlogged packet queue length limitation, and $\sum_{l\in {ℒ}_{c}}Q_{l}^{c}(t)$ denotes the sum of all packets backlogged in the actual queues.

### 2.3. Network Constraints

First, the network remains stable when the backlog of all the queues is less than some finite value [39], which is set as the buffer size constraint $q_{M}$. This constraint can be formulated as

(7) $$\underset{T\to \infty }{\lim\sup}\frac{1}{T}\sum_{t=0}^{T-1}E\{Q_{l}^{c}(t)\}<q_{M},\;\forall c\in F,l\in {ℒ}_{c},$$

(8) $$\underset{T\to \infty }{\lim\sup}\frac{1}{T}\sum_{t=0}^{T-1}E\{X_{c}(t)\}<q_{M},\;\forall c\in F,$$

(9) $$\underset{T\to \infty }{\lim\sup}\frac{1}{T}\sum_{t=0}^{T-1}E\{Y_{c}(t)\}<q_{M},\;\forall c\in F.$$

Second, to maintain the stability of the actual queues, the packet delivery rate of link l should always be larger than that of link l−1. We set a sufficiently small metric $\varepsilon_{l}^{c}$; when flow c is scheduled at link l, its scheduling rate must satisfy the following constraint:

(10) $$\mu_{l}^{c}(t)-\mu_{l-1}^{c}(t)\geq \varepsilon_{l}^{c},\;\forall c\in F,l,(l-1)\in {ℒ}_{c}.$$

When $l=l_{s(c)}$, Equation (10) can be transformed into

(11) $$\mu_{l_{s(c)}}^{c}(t)-R_{c}(t)\geq \varepsilon_{l_{s(c)}}^{c},\;\forall c\in F,l_{s(c)}\in {ℒ}_{c}.$$

The value of $R_{c}(t)$ is constrained by the virtual queue $X_{c}(t)$. To maintain the stability of $X_{c}(t)$, the service rate $R_{c}(t)$ must be bigger than the arrival rate $a_{c}(t)$. Thus, the limitation of the service rate is $0\leq a_{c}(t)\leq R_{c}(t)\leq \mu_{0}$, where $\mu_{0}$ is the maximum throughput rate for the source node.

Third, the scheduling policy of a link is constrained by its interference links, as we mentioned in the link model. All the links working on the same channel in the same area share the capacity of the physical channel [40], which can be expressed as

(12) $$D_{l}^{c}(t)+\sum_{l^{\prime }\in I_{l}}D_{l^{\prime }}^{}=\Gamma_{l},\;\forall c\in F,l\in {ℒ}_{c}$$

where $\Gamma_{l}$ is the capacity of the physical channel on which the links l and $l^{\prime }$ operate. To ensure the scheduling rate of link l, the summed rates of all the interference links should be less than a constant threshold σ:

(13) $$\sum_{l^{\prime }\in I_{l}}D_{l^{\prime }}^{}\leq \sigma ,\;\forall c\in F,l\in {ℒ}_{c}.$$

Finally, according to Little’s Theorem, the average end-to-end delay of flow c can be expressed as

(14) $$\tau_{c}=\frac{\underset{T\to \infty }{\lim\sup}\frac{1}{T}\sum_{t=0}^{T-1}E\{Q_{l}^{c}(t)\}}{\underset{T\to \infty }{\lim\sup}\frac{1}{T}\sum_{t=0}^{T-1}E\{R_{c}(t)\}},\;\forall c\in F,l\in {ℒ}_{c},$$

and $\tau_{c}$ should be no more than the delay threshold $\rho_{c}$, i.e., $\tau_{c}\leq \rho_{c}$.

## 3. Problem Formulation and Lyapunov Drift Optimization

The objective of this paper is to maximize the capacity of the MR-MC WMNs and to ensure the stability of the network while satisfying the constraints of transmission rate, end-to-end delay, and interference issues. The whole problem is formulated as follows:

(15) $$\max\underset{T\to \infty }{\lim}\frac{1}{T}\sum_{t=0}^{T-1}\sum_{c\in F}R_{c}(t)$$

subject to

(15a) $$0\leq a_{c}(t)\leq R_{c}(t)\leq \mu_{0}\leq \mu_{l}^{c}(t),\;\forall c\in F,l\in {ℒ}_{c}$$

(15b) $$\mu_{l}^{c}(t)-\mu_{l-1}^{c}(t)\geq \varepsilon_{l}^{c},\;\forall c\in F,l\in {ℒ}_{c}$$

(15c) $$\sum_{l^{\prime }\in I_{l}}D_{l^{\prime }}^{}\leq \sigma ,\;\forall c\in F,l\in {ℒ}_{c}$$

(15d) $$\tau_{c}\leq \rho_{c},\;\forall c\in F$$

(15e) $$q_{M}\geq 0$$

where the objective function expressed in (15) is to maximize the average service rate of each flow at the source node. Constraint (15a) illustrates the relationship between the arrival rate from the transport layer, the service rate at the source node, the scheduling rate of the source node, and the scheduling rate of any link *l* belonging to the routing path of flow c; (15b) is the scheduling rate constraint of two adjacent links to ensure the stability of each actual queue; (15c) limits the rate of the interference links to guarantee that the current link possesses enough physical channel capacity; and (15d) is the end-to-end delay constraint to satisfy the delay requirements of each flow.

As shown in the queue model in Section 2, all the constraints mentioned above can be transformed into a queue stability problem for all the actual and virtual queues. Let $G(t)=\left\{Q_{l}^{c}(t),Q_{l_{s(c)}}^{c}(t),X_{c}^{}(t),Y_{c}(t)\right\}$ denote the concatenated queue backlog of the network. As $q_{M}$ and $\mu_{0}$ are the crucial metrics for the queue stability, we construct the Lyapunov function as

(16) $$\begin{array}{cc} L(G(t))= & \frac{1}{2}\{\sum_{c\in F}\frac{q_{M}-\mu_{0}}{q_{M}}Q_{l_{s(c)}}^{c}{\left(t\right)}^{2}+\sum_{c\in F}X_{c}{\left(t\right)}^{2}\\ & +\sum_{c\in F}\sum_{l\in {ℒ}_{c}}Y_{c}{\left(t\right)}^{2}+\sum_{c\in F}\sum_{l\in {ℒ}_{c}}\frac{Q_{l_{s(c)}}^{c}(t)}{q_{M}}Q_{l}^{c}{\left(t\right)}^{2}\} \end{array}.$$

Without loss of generality, we assume that all the queues are empty when t=0, such that L(G(0))=0. The conditional Lyapunov drift Δ(G(t)) is defined as

(17) Δ(G(t))=E{L(G(t+1))−L(G(t))|G(t)}.

By employing Equations (2), (4), (5), and (6), we obtain

(18) $$\begin{array}{l} \Delta (G(t))=\frac{1}{2}E\{\sum_{c\in F}\frac{q_{M}-\mu_{0}}{q_{M}}\left(Q_{l_{s(c)}}^{c}{\left(t+1\right)}^{2}-Q_{l_{s(c)}}^{c}{\left(t\right)}^{2}\right)\\ +\sum_{c\in F}\left(X_{c}{\left(t+1\right)}^{2}-X_{c}{\left(t\right)}^{2}\right)+\sum_{c\in F}\sum_{l\in {ℒ}_{c}}\left(Y_{c}{\left(t+1\right)}^{2}-Y_{c}{\left(t\right)}^{2}\right)\\ +\sum_{c\in F}\sum_{l\in {ℒ}_{c}}\left(\frac{Q_{l_{s(c)}}^{c}(t+1)}{q_{M}}Q_{l}^{c}{\left(t+1\right)}^{2}\right)-\sum_{c\in F}\sum_{l\in {ℒ}_{c}}\left(\frac{Q_{l_{s(c)}}^{c}(t)}{q_{M}}Q_{l}^{c}{\left(t\right)}^{2}\right)|G(t)\} \end{array}.$$

Subtracting (15) from (18), the drift-minus-reward term ΔG is obtained as follows:

(19) $$\Delta G=\Delta (G(t))-V\sum_{c\in F}E\left\{R_{c}(t)|G(t)\right\}$$

where V is a nonnegative tunable parameter. For any nonnegative values of x,y, and z, ${\left({\left[x-y\right]}^{+}+Z\right)}^{2}\leq x^{2}+y^{2}+z^{2}-2x(y-z)$ holds [10]. Thus, we obtain the drift-minus-reward ΔG as

(20) $$\begin{array}{cc} \Delta G & =\frac{1}{2}E\{\sum_{c\in F}\frac{q_{M}-\mu_{0}}{q_{M}}\left(D_{l_{s(c)}}^{c}{\left(t\right)}^{2}+R_{c}(t)-2Q_{l_{s(c)}}^{c}(t)D_{l_{s(c)}}^{c}(t)+2Q_{l_{s(c)}}^{c}(t)R_{c}(t)\right)\\ & +\sum_{c\in F}\left(R_{c}{\left(t\right)}^{2}+a_{c}{}^{2}-2X_{c}(t)R_{c}(t)-2X_{c}(t)a_{c}\right)\\ & +\sum_{c\in F}\sum_{l\in {ℒ}_{c}}\left(\rho_{c}{}^{2}R_{c}{\left(t\right)}^{2}+\sum_{l\in {ℒ}_{c}}Q_{l}^{c}{\left(t\right)}^{2}-2Y_{c}(t)\rho_{c}R_{c}(t)+2Y_{c}(t)\sum_{l\in {ℒ}_{c}}Q_{l}^{c}(t)\right)\\ & +\sum_{c\in F}\sum_{l\in {ℒ}_{ℒ}}\left(\frac{Q_{l_{s(c)}}^{c}(t)+R_{c}(t)}{q_{M}}Q_{l}^{c}{\left(t+1\right)}^{2}\right)-\sum_{c\in F}\sum_{l\in {ℒ}_{c}}\left(\frac{Q_{l_{s(c)}}^{c}(t)}{q_{M}}Q_{l}^{c}{\left(t\right)}^{2}\right)|G(t)\} \end{array}$$

(21) $$\begin{array}{cc} \Delta G\leq B & +E\left\{\sum_{c\in F}R_{c}(t)\left(\frac{\left(q_{M}-\mu_{0}\right)Q_{l_{s(c)}}^{c}(t)}{q_{M}}-Y_{c}(t)\rho_{c}(t)-X_{c}(t)-V|G(t)\right)\right\}\\ & +\sum_{c\in F}X_{c}(t)a_{c}+\left|L_{c}\right|q_{M}\sum_{c\in F}Y_{c}(t)+\frac{1}{2}\sum_{c\in F}\frac{\left(2\left|L_{c}\right|-1+\mu_{0}{}^{2}\right)}{q_{M}}Q_{l_{s(c)}}^{c}(t)\\ & -E\{\sum_{c\in F}\frac{\left(q_{M}-\mu_{0}\right)}{q_{M}}Q_{l_{s(c)}}^{c}(t)D_{l_{s(c)}}^{c}(t)\\ & +\sum_{c\in F}\sum_{l\in {ℒ}_{c}}\frac{Q_{l_{s(c)}}^{c}(t)Q_{l}^{c}(t)}{q_{M}}\left(D_{l}^{c}(t)-D_{l-1}^{c}(t)\right)|G(t)\} \end{array}$$

where $\left|{ℒ}_{c}\right|$ is the number of hop counts of flow c, and |F| is the number of all flows. B is a positive constant constrained by the following equation:

(22) $$B\geq \frac{1}{2}\left|{ℒ}_{c}\right|\left|F\right|q_{M}\mu_{0}+\left|F\right|\frac{q_{M}-\mu_{0}}{q_{M}}\mu_{0}{}^{2}+\frac{1}{2}\mu_{0}{}^{2}\sum_{c\in F}\rho_{c}{}^{2}+\frac{1}{2}\left|F\right|{\left|{ℒ}_{c}\right|}^{2}q_{M}{}^{2}+\frac{1}{2}\left|F\right|\mu_{0}{}^{2}+\frac{1}{2}\left|F\right|a_{c}{}^{2}.$$

The last term of the right-hand side (RHS) in (20) can be rewritten by simple algebra as in [38].

(23) $$\begin{array}{cc} -E & \{\sum_{c\in F}\frac{\left(q_{M}-\mu_{0}\right)}{q_{M}}Q_{l_{s(c)}}^{c}(t)D_{l_{s(c)}}^{c}(t)+\sum_{c\in F}\sum_{l\in {ℒ}_{c}}D_{l}^{c}(t)\frac{Q_{l_{s(c)}}^{c}(t)}{q_{M}}\left(D_{l-1}^{c}(t)-D_{l}^{c}(t)\right)\\ & +\sum_{c\in F}D_{l_{s(c)}}^{c}(t)\frac{Q_{l_{s(c)}}^{c}(t)}{q_{M}}(q_{M}-\mu_{0}-Q_{l_{s(c)}}^{c}(t))|G(t)\} \end{array}$$

The second term of the RHS in (21) is maximized by the service rate $R_{c}(t)$. The last term of the RHS in (21) is optimized by the transmission rate $\mu_{l}^{c}(t)$, while (23) is optimized by the scheduling policy $D_{l}^{c}(t)$. Thus, the original stochastic optimization problem in (15) can be transformed into a series of successive instantaneous static optimization problems.

## 4. The Distributed Rate-Control and Delay-Aware Scheduling Algorithm

Now we propose a distributed rate-control and delay-aware scheduling algorithm (DRDA) for the MR-MC multi-hop WMNs with multicommodity flows which can stabilize the network while satisfying the arrival data rate and delay constraints of each flow. The scheduling policy of a flow in the scheduling controller is calculated by the DRDA algorithm, which consists of two parts: scheduling decision and rate control.

### 4.1. Scheduling Decision

The scheduling decision $\delta_{l}^{c}(t)$ of link l with flow c can be calculated as

(24) $$\delta_{l}^{c}(t)\left\{ \begin{array}{l} 1,\;c=c^{*}\\ 0,\;otherwise \end{array}\right.$$

(25) $$c^{*}=\arg\underset{c\in F}{\;\max}\omega_{l}^{c}(t),\;\forall c,c^{*}\in F,l\in {ℒ}_{c}$$

where $\omega_{l}^{c}(t)$ is the flow weighted metric which is calculated as follows.

(26) $$\omega_{l}^{c}(t)\left\{ \begin{array}{l} \frac{Q_{l_{s(c)}}^{c}(t)}{q_{M}}\left(D_{l-1}^{c}(t)-D_{l}^{c}(t)\right),\;\forall c\in F,l\in {ℒ}_{c},l\neq l_{s(c)}\\ \frac{Q_{l_{s(c)}}^{c}(t)}{q_{M}}(q_{M}-\mu_{0}-Q_{l_{s(c)}}^{c}(t)),\;\forall c\in F,l\in {ℒ}_{c},l=l_{s(c)} \end{array}\right.$$

Parameter $\omega_{l}^{c}(t)$ is calculated in the scheduling controller in turn by (26) based only on local information. The flow with the largest weight will be selected as the candidate flow $c^{*}$ for scheduling.

### 4.2. Rate Control

According to the second and last terms of the RHS in (21), the rate control can be divided into the service rate control at the source node of flow *c* and the scheduling rate control of each link of flow *c*. The optimization problem of service rate $R_{c}(t)$ is expressed as

(27) $$\underset{a_{c}\leq R_{c}(t)\leq \mu_{0}(t)}{\min}R_{c}(t)\left(\frac{\left(q_{M}-\mu_{0})Q_{l_{s(c)}}^{c}(t\right)}{q_{M}}-X_{c}(t)-Y_{c}(t)\rho_{c}-V\right),\;\forall c\in F,l,l_{s(c)}\in {ℒ}_{c}.$$

The corresponding solution is

(28) $$R_{c}(t)\left\{ \begin{array}{l} \mu_{0}(t),\;\left(\frac{\left(q_{M}-\mu_{0})Q_{l_{s(c)}}^{c}(t\right)}{q_{M}}-X_{c}(t)-Y_{c}(t)\rho_{c}-V\right)<0\\ a_{c}(t),\;otherwise \end{array}\right.$$

where *V* > 0 is a control parameter. It can be easily observed from (28) that when *V* is sufficiently large, the optimal value of $R_{c}(t)$ is $\mu_{0}(t)$; when *V* is small, the optimal value of $R_{c}(t)$ is $a_{c}(t)$.

The optimal scheduling rate $\mu_{l}^{c}(t)$ of each link can be obtained by solving a maximization problem with the link rate constraints which were illustrated in Section 2. The new problem is expressed as

(29) $$\underset{D_{l_{s(c)}}^{c}(t),D_{l}^{c}(t)}{\max}\left(\sum_{c\in F}\frac{\left(q_{M}-\mu_{0}\right)}{q_{M}}Q_{l_{s(c)}}^{c}(t)D_{l_{s(c)}}^{c}(t)+\sum_{c\in F}\sum_{l\in {ℒ}_{c}}\frac{Q_{l_{s(c)}}^{c}(t)Q_{l}^{c}(t)}{q_{M}}\left(D_{l}^{c}(t)-D_{l-1}^{c}(t)\right)\right)$$

subject to

(29a) $$\mu_{l}^{c}(t)-\mu_{l-1}^{c}(t)\geq \varepsilon_{l}^{c},\;\forall c\in F,l,(l-1)\in {ℒ}_{c}$$

(29b) $$\mu_{l_{s(c)}}^{c}(t)-R_{c}(t)\geq \varepsilon_{l_{s(c)}}^{c},\;\forall c\in F,l_{s(c)}\in {ℒ}_{c}$$

(29c) $$\varepsilon_{\min}\leq \varepsilon_{l}^{c}(t),\varepsilon_{l_{s(c)}}^{c}(t)\leq \varepsilon_{\max},\;\forall c\in F,l,l_{s(c)}\in {ℒ}_{c}$$

(29d) $$D_{l}^{c}(t)+\sum_{l^{\prime }\in I_{l}}D_{l^{\prime }}^{}=\Gamma_{l},\;\forall c\in F,l\in {ℒ}_{c}$$

(29e) $$\sum_{l^{\prime }\in I_{l}}D_{l^{\prime }}^{}\leq \sigma ,\;\forall c\in F,l\in {ℒ}_{c}$$

where $\varepsilon_{\min}$ should be sufficiently small and $\varepsilon_{\max}$ is a global value which will be discussed later. We assume that for all the scheduling policies $\delta_{l}^{c}(t)=1$; then problem (29) can be transformed into

(30) $$\underset{D_{l_{s(c)}}^{c}(t),D_{l}^{c}(t)}{\max}\left(\sum_{c\in F}\frac{\left(q_{M}-\mu_{0}\right)}{q_{M}}Q_{l_{s(c)}}^{c}(t)D_{l_{s(c)}}^{c}(t)+\sum_{c\in F}\sum_{l\in {ℒ}_{c}}\frac{Q_{l_{s(c)}}^{c}(t)Q_{l}^{c}(t)}{q_{M}}\left(D_{l}^{c}(t)-D_{l-1}^{c}(t)\right)\right)$$

subject to

(29a)-(29c) hold

(30a) $$\mu_{l}^{c}(t)+\sum_{l^{\prime }\in I_{l}}\mu_{l^{\prime }}^{}=\Gamma_{l},\;\forall c\in F,l\in {ℒ}_{c}$$

(30b) $$\sum_{l^{\prime }\in I_{l}}\mu_{l^{\prime }}^{}\leq \sigma ,\;\forall c\in F,l\in {ℒ}_{c}.$$

The constraints (30a) and (30b) can only be satisfied in a centralized way, which is inefficient for MR-MC multi-hop WMNs with multicommodity flows. Therefore, we transform the centralized conditions to distributed ones. Assuming that $R_{c}(t)$ is equal to its optimal solution $\mu_{0}$, then $\mu_{l}^{c}(t)$ can be rewritten as

(31) $$\begin{array}{cc} \mu_{l}^{c}(t) & =\mu_{l_{s(c)}}^{c}(t)+\sum_{l^{*}:h_{l^{*}}\in [1,h_{l}]}\varepsilon_{l^{*}}\\ & =\mu_{0}+\varepsilon_{l_{s(c)}}^{c}(t)+\sum_{l^{*}:h_{l^{*}}\in [1,h_{l}]}\varepsilon_{l^{*}}\\ & \leq \mu_{0}+h_{l}\varepsilon_{\max} \end{array}.$$

For each interference link, we have

(32) $$\mu_{l^{\prime }}^{c}(t)\leq \mu_{0}+h_{l^{\prime }}\varepsilon_{\max},\;\forall c\in F,l^{\prime }\in I_{l},l\in {ℒ}_{c}.$$

In the following, we introduce a distributed rate control Algorithm 1 to get the global value $\varepsilon_{\max}$ and the optimal scheduling rate $\mu_{l}^{c}(t)$. The specific steps are as follows:

Step 1: After the routing detection, every node can get the information of the channel strategy and interference from neighbor nodes in a distributed way. When the flow traverses link l, all the local information is sent to the source node and $\varepsilon_{\max}^{c}$ can be calculated locally according to (31) and (32).

Step 2: The minimum-consensus algorithm [37] is used to iteratively calculate the value of $\varepsilon_{\max}$ in a distributed way through all the $\varepsilon_{\max}^{c}$ of the source nodes. In each iteration, every source node broadcasts its $\varepsilon_{\max}^{c}$ to its immediate neighbors, then compares the received $\varepsilon_{\max}^{c}$ from other nodes with its own $\varepsilon_{\max}^{c}$ value and updates $\varepsilon_{\max}^{c}$ to the smaller one. Other non-source nodes only need to forward the smallest received $\varepsilon_{\max}^{c}$ to their neighbors. The algorithm will finally achieve global convergence when $\varepsilon_{\max}$ is equal to the smallest $\varepsilon_{\max}^{c}$ of all the source nodes.

Step 3: After getting $\varepsilon_{\max}$, the source nodes of each flow use (30b) to calculate the scheduling rate $\mu_{l}^{c}(t)$ of each link included in the flow’s routing path and then transmit them along each hop to the corresponding links.


**Algorithm 1: Distributed Rate Control**
1: Each node gets its channel strategy and interference nodes through routing detection.2: **for**
c=1…|F|3:  **for**
$n=1\ldots \left|N_{c}\right|$4:    Send local information to its source node;5:  **end**6:   Calculate $\varepsilon_{\max}^{c}$ in the source node;7: **end**8: Update $\varepsilon_{\max}^{c}$ through minimum-consensus algorithm.9: **for**
c=1…|F|10:   Calculate $\mu_{l}^{c}(t)$;11:   Send $\mu_{l}^{c}(t)$ to each node;12: **end**

**Theorem** **1.** *Given that*$\rho_{c}\geq \frac{\left|ℒ\right|q_{M}}{r_{c,\xi }^{*}}$and$q_{M}\geq \frac{2\left|ℒ\right|-1+\mu_{0}{}^{2}}{2\xi }+\mu_{0}$, *the DRDA algorithm can achieve a time average throughput of*

(33) $$\underset{t\to \infty }{\lim\;\inf}\frac{1}{t}\sum_{\tau =0}^{t-1}\sum_{c\in F}E\{R_{c}(t)\}\geq \sum_{c\in F}r_{c,\delta }^{*}-\frac{B}{V}$$

(34) $$B=\left|F\right|\frac{q_{M}-\mu_{0}}{q_{M}}\mu_{0}{}^{2}+\frac{1}{2}\left(\left|{ℒ}_{c}\right|\left|F\right|q_{M}+\mu_{0}\mu_{0}{}^{2}\sum_{c\in F}\rho_{c}{}^{2}+\left|F\right|{\left|{ℒ}_{c}\right|}^{2}q_{M}{}^{2}+\left|F\right|\mu_{0}{}^{2}+\left|F\right|a_{c}{}^{2}\right).$$

*In addition, DRDA ensures an upper bound of the virtual queue backlogs of*

(35) $$\underset{t\to \infty }{\lim\;\inf}\frac{1}{t}\sum_{\tau =0}^{t-1}\sum_{c\in F}E\{Q_{l_{s(c)}}^{c}(\tau {)+X}_{c}(\tau {)+Y}_{c}(\tau )\}\leq \frac{B+VK\mu_{0}}{\theta }.$$

**Proof** **of** **Theorem** **1.** Before the proof, we introduce some auxiliary variables. We define Λ as the capacity region consisting of all the available service rates $R_{c}(t)$. Let $r_{c}$ represent the time-average value of $R_{c}(t)$ and let $r_{c}^{*}\in \Lambda$ be the optimal solution of the following optimization problem:

(36) $$\underset{r_{c}:(r_{c}+\xi )\in \Lambda }{\max}\sum_{c\in F}r_{c,}\;\forall c\in F$$

where $r_{c}^{}$ should satisfy $r_{c}^{}+\xi \in \Lambda$, and ξ is a positive number that can be chosen to be arbitrarily small. Define $r_{c,\xi }^{*}=r_{c}^{*}+\xi$ and $r_{c,2\xi }^{*}=r_{c}^{*}+2\xi$, which satisfies $r_{c,\xi }^{*},\;r_{c,2\xi }^{*}\in \Lambda$. When ξ→0, we have

(37) $$\underset{\xi \to 0}{\lim}\sum_{c\in F}r_{c,\xi }^{*}=\sum_{c\in F}r_{c}^{*},\;\forall c\in F.$$

By substituting $r_{c,\xi }^{*}$ and $r_{c,2\xi }^{*}$ into the second term and the last term of RHS of (21), respectively, the drift-minus-reward ΔG can be rewritten as

(38) $$\begin{array}{cc} \Delta G & =\Delta (t)-V\sum_{c\in F}E\left\{R_{c}(t)|G(t)\right\}\\ & \leq B-V\sum_{c\in F}r_{c,\xi }^{*}-\sum_{c\in F}\frac{Q_{l_{s(c)}}^{c}}{q_{M}}\left(\xi \left(q_{M}-\mu_{0}\right)-\frac{\left(2\left|{ℒ}_{c}\right|-1+\mu_{0}{}^{2}\right)}{2}\right)\\ & -\sum_{c\in F}\left(r_{c,\xi }^{*}-a_{c}\right)X_{c}(t)-\sum_{c\in F}\left(\rho_{c}r_{c,\xi }^{*}-\left|{ℒ}_{c}\right|q_{M}\right)Y_{c}(t) \end{array}.$$

We define $\xi_{1}$, $\xi_{2}$, and $\xi_{3}$ satisfying $0<\xi_{1}\leq \rho_{c}r_{c,\xi }^{*}-\left|ℒ\right|q_{M}$, 0<ξ2≤(ξ(qM−μ0)−|ℒ|+12−μ022)/qM, and $0\leq \xi_{3}\leq r_{c,\xi }^{*}-a_{c}$. We can find $\xi =\min\{\xi_{1},\xi_{2},\xi_{3}\}$ such that

(39) $$\begin{array}{l} \Delta (t)-V\sum_{c\in F}E\left\{R_{c}(t)|G(t)\right\}\\ \leq B-\xi \sum_{c\in F}\left(Q_{l_{s(c)}}^{c}(t)+X_{c}(t)+Y_{c}(t)\right)-V\sum_{c\in F}r_{c,\xi }^{*} \end{array}.$$

By taking the expectation with respect to the distribution of G on both sides of (39) and taking the time average on τ=0,1,…,t−1, we can get

(40) $$\begin{array}{l} \frac{1}{t}E\left\{L(G(t))\right\}-\frac{V}{t}\sum_{\tau =0}^{t-1}\sum_{c\in F}E{\{R}_{c}(\tau )\}\\ \leq B-V\sum_{c\in F}r_{c,\xi }^{*}-\frac{\xi }{t}\sum_{\tau =0}^{t-1}\sum_{c\in F}E\{Q_{l_{s(c)}}^{c}(\tau {)+X}_{c}(\tau {)+Y}_{c}(\tau )\} \end{array}.$$

Taking the lim inf of t in (40), we have

(41) $$\begin{array}{l} \underset{t\to \infty }{\lim\;\inf}\frac{1}{t}E\left\{L(G(t))\right\}-\underset{t\to \infty }{\lim\;\inf}\frac{V}{t}\sum_{\tau =0}^{t-1}\sum_{c\in F}E{\{R}_{c}(\tau )\}\\ \leq B-V\sum_{c\in F}r_{c,\xi }^{*}-\underset{t\to \infty }{\lim\;\inf}\frac{\xi }{t}\sum_{\tau =0}^{t-1}\sum_{c\in F}E\{Q_{l_{s(c)}}^{c}(\tau {)+X}_{c}(\tau {)+Y}_{c}(\tau )\} \end{array}.$$

When the term in (41) is nonnegative, we can obtain (33) and (35). □

## 5. Simulation Results

In this section, the performance of the proposed algorithm is evaluated via simulations done in Network Simulator 2 (NS2). We consider an MR-MC WMN with 30 static nodes which are randomly and uniformly distributed in a square of 1000 m×1000 m. The transmission and interference ranges of each node were both set to 250 m. We randomly chose five pairs of source and destination nodes to constitute five flows with $a_{{}_{c}}=1\mathrm{Mbps},\;\rho_{c}=0.5s$. The maximum number of radios on each node was three, the carrier frequency was 2.4 GHz, and there were four available channels, each with a bandwidth of 5 MHz. In the simulations, ad hoc on-demand multi-path distance vector routing (AOMDV) and user datagram protocol (UDP) were adopted as the routing and transfer protocal, respectively. The parameter configuration in the NS2 simulations is shown in Table 2.

**Figure 3:**
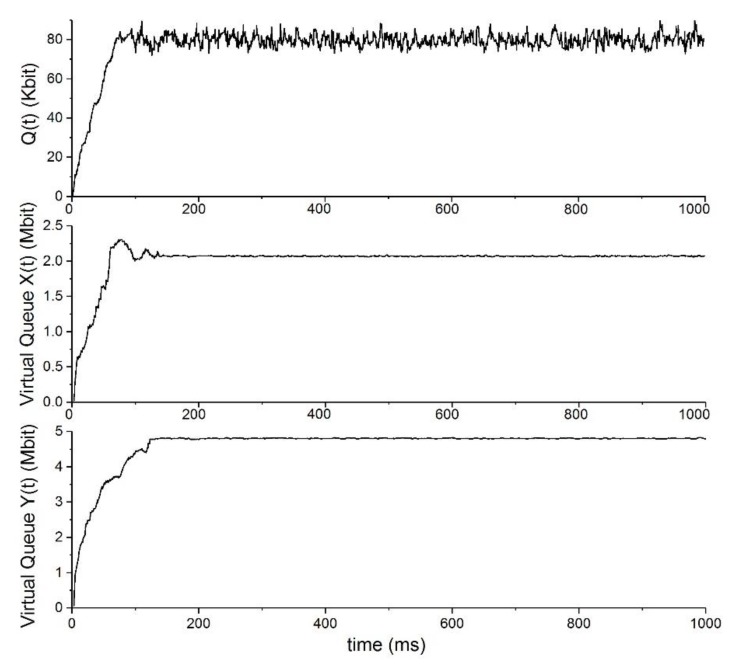
Queue stability.

Figure 3 demonstrates the stability of the actual and virtual queues with $V=1000,\;q_{M}=5\;\mathrm{Mbit}$ settings. We randomly selected a source node to record its virtual queues $X_{c}(t)$ and $Y_{c}(t)$. It can be seen that the backlog of all the virtual queues converged to a stable value which was strictly lower than the maximum buffer size $q_{M}$, as we discussed in Section 4. The behavior of the actual queue $Q_{l}^{c}(t)$ was observed by randomly choosing a non-source node. It can be seen that although the backlog of the actual queue did not converge to a constant, it fluctuated slightly around a fixed value.

**Figure 4:**
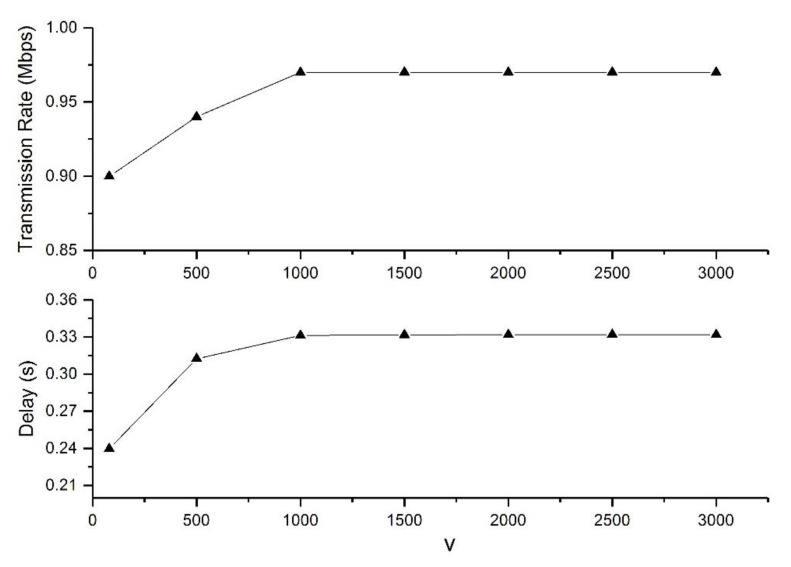
Network performance of the distributed rate-control and delay-aware (DRDA) algorithm versus the value of *V*.

Figure 4 shows the average transmission rate and delay of all the data flows versus parameter *V* with $q_{M}=5\;\mathrm{Mbit}$. It was observed that with increasing *V*, the average transmission rate and end-to-end delay both firstly increased, and they remained stable when *V* ≥ 1000. The maximum delay was still less than the limit value $\rho_{c}=0.5\;s$. Thus, we usually took *V* = 1000 in the simulations.

**Figure 5:**
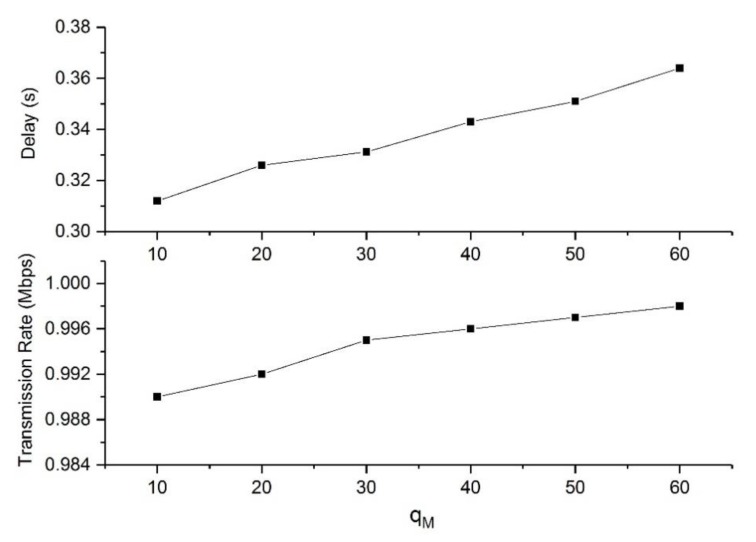
Network performance of DRDA versus the value of $q_{M}$.

Figure 5 shows the network performance of DRDA versus the maximum buffer size $q_{M}$ with *V* = 1000. The average transmission rate of the flow was not sensitive to changes in the value of $q_{M}$. However, the average end-to-end delay increased with $q_{M}$ in an obvious way. This is because the larger the $q_{M}$, the more data will be admitted into the data queue for transmission, resulting in an increasing number of backlogged packets. However, the backlogged packets can only cause an increase in the transmission rate in a very short time. When the backlogs of all queues converge to a stable value, the transmission rate will decrease rapidly.

**Figure 6:**
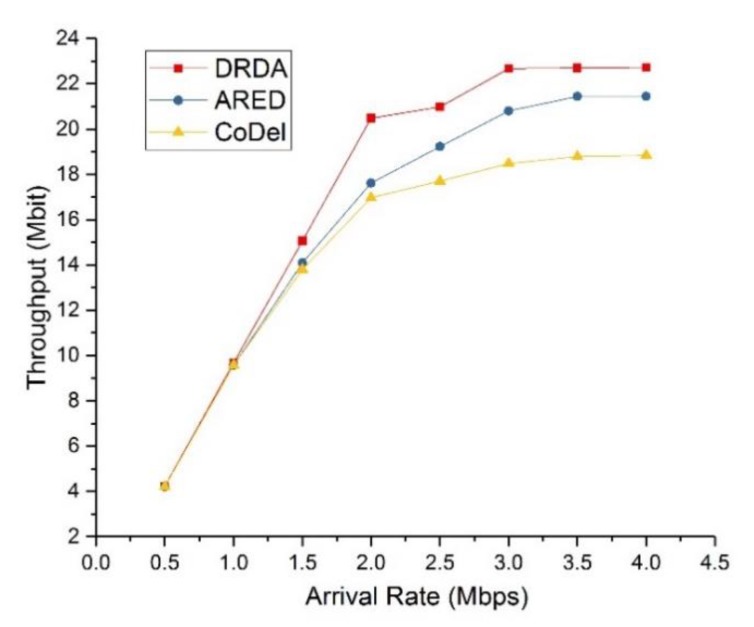
Throughput of DRDA compared with that of ARED and CoDel.

**Figure 7:**
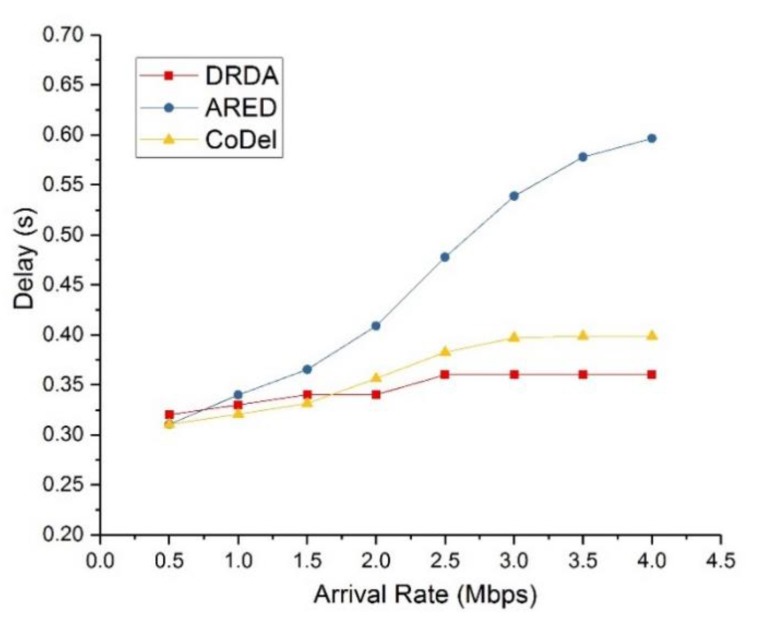
End-to-end delay of DRDA compared with that of ARED and CoDel.

Finally, we compared the performance of DRDA with that of two relevant algorithms. One of the comparison algorithms is advanced random early detection (ARED) [41], which drops the backlogged packets according to a probability calculated based on the average queue length and bandwidth of links. The other comparison algorithm is controlling queue delay (CoDel) [42], which aims to keep queue delay low by dropping some of the backlogged packets. Figure 6 and Figure 7 depict the throughput and end-to-end delay, respectively, of the different algorithms with varying values of the data arrival rate $a_{c}$. The control parameter and buffer size were set to *V* = 1000 and $q_{M}=5Mbit$.

In Figure 6, we demonstrate the network throughput within a period of two seconds. Here, the total number of packets received by the destination nodes was used to represent the network throughput. It was observed that the performance of DRDA was better than that of the other two comparison algorithms. Meanwhile, the throughput of DRDA no longer increased when the arrival rate was such that $a_{c}\geq \mu_{0}$. The throughput of CoDel grows very slowly when the arrival rate is large; this is because too many backlogged packets are dropped to ensure low end-to-end delay.

In Figure 7, the end-to-end delay of DRDA is compared with that of ARED and CoDel. It was found that algorithms DRDA and CoDel can always satisfy the end-to-end flow delay requirement. However, with increasing arrival rate, the delay of ARED increases rapidly, even exceeding the delay constraint. This is because the aim of ARED is to ensure the fairness of all the packets, rather than meeting the delay requirement.

## 6. Conclusions

In this paper, a distributed rate-control and delay-guaranteed scheduling algorithm (DRDA) for MR-MC WMNs was proposed in which the scheduling policy and service rate are optimized through the Lyapunov drift optimization technique. The scheduling policy and scheduling rate of each flow can be decided based only on local information. The simulation results showed that our proposed algorithm can maintain the stability of all the actual and virtual queues while maximizing the network throughput and meeting the delay constraint of each flow as desired.

## Figures and Tables

**Table 1:** Summary of key notation.

Notation	Meaning
N	Set of nodes in the network
${ℒ}_{p}$	Set of physical links
T	Set of tuples
ℒ	Set of tuple-links
${ℒ}_{c}$	Set of tuple-links with flow c
$N_{c}$	Set of nodes with flow c
$r_{n}$	Set of radios in node n
e	Set of channels
$e_{r_{n}}$	The channel which radio $r_{n}$ operates on
$I_{l}$	Set of interference links of link l
F	Set of all flows in the network
$F_{s}$	Set of flows which are generated in node s
$a_{c}$	The minimum arrival data rate of flow c
$\rho_{c}$	The delay constraint of flow c
$Q_{l}^{c}(t)$,	Queue length of link l with flow c
$Q_{l_{s(c)}}^{c}(t)$	Queue length of the source link with flow c
$D_{l}^{c}(t)$	Scheduling policy of link l with flow c
$\delta_{l}^{c}(t)$	Scheduling weighted metric of link l with flow c
$\mu_{l}^{c}(t)$	Scheduling rate of link l with flow c
$R_{c}(t)$	Service rate of flow c at its source node
$X_{c}(t)$, $Y_{c}(t)$	Backlog of virtual queues *X* and *Y* with flow c
$\mu_{0}$	Maximum rate of each source node
$q_{M}$	Maximum buffer size of the queue

**Table 2:** Parameter configuration in Network Simulator 2 (NS2) simulations.

Channel Type	Wireless Channel
Propagation Model	Two Ray Ground
Antenna Height	2 m
Working Frequency	2.4 GHz
Single Channel Bandwidth	5 MHz
Communication Distance	250 m
Interference Distance	250 m
Routing Protocol	AOMDV
Data Type	Constant Bate Rate (CBR)
Transfer Protocol	UDP
Maximum Number of Radios	3
$a_{{}_{c}}$	1 Mbps
$\rho_{c}$	0.5 s
V	1000
